# Metabolomics analyses of serum metabolites perturbations associated with *Naja atra* bite

**DOI:** 10.1371/journal.pntd.0011507

**Published:** 2023-08-28

**Authors:** Dongling He, Shaocong Hu, Zhi Huang, Caifeng Mo, Xiaoyang Cheng, Pengshu Song, Yalan Li, Tianlin Song, Zhezhe Guan, Yi Zhou, Xuerong Zhang, Ming Liao

**Affiliations:** Life Science Institute Guangxi Medical University, Nanning, PR China; Instituto Butantan, BRAZIL

## Abstract

*Naja atra* bite is one of the most common severe snakebites in emergency departments. Unfortunately, the pathophysiological changes caused by *Naja atra* bite are unclear due to the lack of good animal models. In this study, an animal model of *Naja atra* bite in Guangxi *Bama miniature pigs* was established by intramuscular injection at 2 mg/kg of *Naja atra* venom, and serum metabolites were systematically analyzed using untargeted metabolomic and targeted metabolomic approaches. Untargeted metabolomic analysis revealed that 5045 chromatographic peaks were obtained in ESI+ and 3871 chromatographic peaks were obtained in ESI-. Screening in ESI+ modes and ESI- modes identified 22 and 36 differential metabolites compared to controls. The presence of 8 core metabolites of glutamine, arginine, proline, leucine, phenylalanine, inosine, thymidine and hippuric acid in the process of *Naja atra* bite was verified by targeted metabolomics significant difference (P<0.05). At the same time, during the verification process of the serum clinical samples with *Naja atra* bite, we found that the contents of three metabolites of proline, phenylalanine and inosine in the serum of the patients were significantly different from those of the normal human serum (P<0.05). By conducting functional analysis of core and metabolic pathway analysis, we revealed a potential correlation between changes in key metabolites after the *Naja atra* bite and the resulting pathophysiological alterations, and our research aims to establish a theoretical foundation for the prompt diagnosis and treatment of *Naja atra* bite.

## Author summary

*Naja atra* bite is one of the most serious tropical and subtropical diseases, and the pathophysiological mechanism of injury caused by *Naja atra* bite is still not clear, meanwhile, large animal models of *Naja atra* bite are rarely reported. In this study, a *Naja atra* bite *Bama miniature pig* model was constructed and evaluated. In addition, 22 and 36 metabolites were found to be significantly altered in ESI+ and ESI- mode by untargeted metabolomics assay, respectively. More interestingly, 8 of them co-occurred in both ESI+ and ESI- modes. We quantified these 8 metabolites absolutely by targeted metabolomics and validated them in the serum of *Naja atra* bite patients, which showed the same trend as the untargeted metabolomics results. We also compared the differences in the levels of these 8 metabolites in the serum of the *Naja atra* bite patient group and the normal human group, we found decreased expression of inosine, proline and phenylalanine. Based on this, we compared the blood metabolic profiles with those of *Bungarus multicinctus* and *Trimeresurus stejnegeri*, and finally we confirmed that inosine, proline and phenylalanine are likely to be the key metabolites in *Naja atra* bite.

## Introduction

Snakebites are classified as one of the important tropical diseases by the World Health Organization that are easily neglected [1], with approximately 5 million snakebites occurring each year, resulting in 25,000 to 125,000 deaths all over the world [2,3]. As many as 300,000 patients suffer from snakebites each year in China, 2/3 of them are young and middle-aged, with a mortality rate of 5% to 10% and 25% to 30% of those disabled or incapacitated, the occurrence of snakebites places a great burden on their families [4]. *Naja atra* also known as Zhoushan cobra, is one of the most common critical illnesses in the emergency department and has long ranked among the top three snakebites in China [5,6]. Unfortunately, *Naja atra* bite act very rapidly on the organism, with rapid local tissue edema occurring in about 10 min, which can easily lead to ulcer formation or massive local necrosis if not treated properly, and it can lead to disability or even amputation in severe cases [7].

Studies have shown that the venom of *Naja atra* is a complex mixture of cytotoxin, neurotoxin, phospholipase, hyaluronidase and proteolytic enzyme, which can damage the muscle tissue, nervous system, blood system, digestive system and endocrine system [8,9]. Among them, cytotoxin, proteolytic enzyme, phospholipase and hyaluronidase can cooperate with each other in the cell membrane, lysosome, mitochondria and other binding targets, resulting in cell swelling, rupture and death. It can further dissolve muscle tissue, destroy capillary endothelium and basement membrane as the disease progresses, subsequently release vasoactive substances such as epinephrine, serotonin, and histamine. It causes damage to muscle tissue and blood vessel wall, causing necrosis and bleeding of local bite tissue, anleadn leads to deep tissue ulceration and tissue necrosis. In severe cases, large necrosis deep into muscle fascia and periosteum can occur, resulting in disability of affected limbs [10–14]. The neurotoxin in the venom can cause neuromuscular blockade to the body, which can lead to the loss of motor function of skeletal muscles and various kinds of paralysis. Its early clinical manifestations are blepharoptosis and dysphagia, there may be respiratory paralysis, respiratory failure, respiratory arrest and so on [15,16].

Although we have some understanding of the mechanism of underlying intoxication and pathophysiological changes caused by *Naja atra* bite, the rapid and accurate diagnosis of *Naja atra* bite is one of the major clinical problems currently faced due to the lack of appropriate animal models. Metabolomics is the science that studies about the types and quantities of metabolites and their changing patterns after an organism is perturbed, and also reflects the series of biological events that occur in a certain pathophysiological process by revealing the overall trajectory of metabolism under the influence of intrinsic and extrinsic factors. This is one of the most important means of finding potential biomarkers. We previously reported that we have used *Bama miniature pigs* as an animal model, to explore the changes of metabolites and analyze the potential biomarkers and metabolic pathways after the *Bungarus multicinctus* bite and *Trimeresurus stejnegeri* bite [17,18]. In the present study, we still used an animal model of *Naja atra* bite in *Bama miniature pigs* to screen, identify and validate the differential metabolites using untargeted and targeted metabolomics techniques. Additionally, we performed bioinformatics analysis of the obtained differential metabolites. Furthermore, serum samples were collected from patients, and the trends of the identified key metabolites in serum were validated using targeted metabolomics. In conclusion, our study successfully constructed a large animal model for *Naja atra* bite, supplanting the previous utilization of small animal models. Our study aims to investigate the alterations in organismal metabolites following a *Naja atra* bite and identify potential biomarkers associated with *Naja atra* envenomation. By doing so, we seek to offer essential theoretical support for the clinical diagnosis, treatment, and understanding of the pathophysiological changes induced by *Naja atra* bite.

## Methods and materials

### Ethics statement

The relevant materials of human biomedical research involved in this experiment were reviewed and approved by the Medical Ethics Committee of Guangxi Medical University. Animal experiments were approved by the Experimental Animal Ethics Committee of Guangxi Medical University. The human blood samples were used after the patients agreed and signed the informed consent form approved by Guangxi Medical University.

### Experimental animals and husbandry environment

Fifty Kunming (KM) mice (18–22 g, half male and half female) were purchased from Guangxi Medical University Laboratory Animal Center (Guangxi, China, approval No. SCXK 【Gui】2018–0003). The mice were kept in standard SPF grade laboratories (temperature 20°C-25°C, relative humidity 50%-65%, free access to water) and fed ordinary feed three times a day for one week. Twenty-six Guangxi *Bama miniature pigs* (12 kg, 3–4 months old, half male and half female) were purchased from the Experimental Animal Center of Guangxi University (Guangxi, China, approval No. SCXK 【Gui】2018–0003). The pigs were kept in the general laboratory (temperature 20°C-25°C, free access to water) and fed ordinary feed two times a day. Animal experiments were performed according to the protocol approved by the Experimental Animal Welfare and Ethics Committee of Guangxi Medical University.

### Main experimental reagents and materials

*Naja atra* venom lyophilized powder was obtained from the Snake Venom Research Institute of Guangxi Medical University and stored at -80°C. Ultrapure water was filtered by Milli-Q (USA) and then used. Formic acid (HPLC purity) and acetonitrile (HPLC purity) were purchased from Merck (Germany). ammonium acetate (HPLC purity), inosine standard, horse uric acid standard, goose deoxycholic acid standard, thymidine standard, glycocholic acid, glutamine standard, leucine standard, phenylalanine standard, proline standard and arginine standard were purchased from Sigma (USA). Serum samples from patients bitten by Naja atra were provided by the Emergency Department of the First Clinical Medical College of Guangxi Medical University and the Emergency Department of the Second Clinical Medical College of Guangxi Medical University.

### Establishment and validation of a model for *Naja atra* venom injection in *Bama miniature pigs*

#### Explore the LD_50_ of *Naja atra* venom injected mice and estimate the LD_50_ of *Naja atra* venom-injected *Bama miniature pigs*

Fifty KM mice were randomly divided into 5 groups. The venom was configured into five dose groups of 1.0, 0.90, 0.81, 0.73 and 0.65 mg/kg using the isometric dilution method at 0.9 group spacing. Mice were observed to die within seven days after injection. According to the LD_50_ of *Naja atra* venom injected intramuscularly in mice, the LD_50_ of *Naja atra* venom injected intramuscularly in *Bama miniature pigs* was calculated by the equivalent volume conversion algorithm.

#### Construction of a *Bama miniature pig* model for *Naja atra* venom injection

Six pigs were divided into two groups (half male and half female) and fasted for 12h before the experiment. The pigs in the venom group were injected with snake venom solution (2mg/kg, 10 mg/mL, 3 ml) at the muscle of the leg, and the pigs in the control group were injected with the same volume of saline at the same site. During the experiment, we observed the biological behavioral status of the pigs such as mental status, locomotor status, and wound changes.

#### Hematological index examination and HE staining

Blood was collected from every six pigs using blood collection tubes containing EDTA before injection and 3h, 6h and 12h after injection. The blood was analyzed by an automatic blood analyzer (BC-5390, Shenzhen, China) for routine blood tests, an automatic coagulation analyzer (CS-5100, Sysmex, Japan) for PT, APTT, TT and FIB, and automatic biochemical analyzer (BS-2000, Shenzhen, China) for biochemical blood levels. The pigs were anesthetized, then the brain tissue, heart muscle, liver and muscle at the injection site of the experimental group and the control group were stained with HE. Each tissue was cut into 1.5 cm^3^ size and placed in 4% paraformaldehyde solution for 48h. Pathological sections were made by dehydration, embedding, sectioning, dehydration and staining, and histopathological changes were observed under the light microscope.

#### Sample collection and pretreatment for Metabolomics experiments

Twenty pigs were randomly divided into two groups (half male and half female) and intramuscular injection was used as before. After 6h of injection, blood samples were taken from each group of pigs in turn, centrifuged twice (4°C, 3000rcf, 10min), the supernatant was mixed with pre-cooled methanol acetonitrile solution (V_methanol_/V_acetonitrile_ = 1:1) in the ratio of 100μL:400μL, vortexed and shaken for 60s, then the Metabolites were precipitated by resting at -20°C for 2h and centrifuged (15000rcf, 4°C, 20min). The Metabolites were re-solubilized with 120 μL of the mixture (95% water+5% Acetonitrile) before sampling for mass spectrometry and filtered using a 0.22 μm microporous membrane.

### Untargeted metabolomics analysis

#### UPLC-Q/TOF-MS conditions

The sample was separated by an Agilent 1290 Infinity LC ultra-high performance liquid chromatography system hilic column, and the separation conditions are shown in Table 1.

**Table 1:** HPLC conditions.

Project	Condition
Column temperature	25°C
Flow rate	0.3 ml/min
Mobile phase A	H2O + 25mM CH3COONH4 + 25mM NH3 ·H2O
Mobile phase B	CH3CN
Gradient elution procedure	0–1 min, B, 95%; 1–14 min, B, 95–65%; 14–16 min, B, 65%-40%; 16–18 min, B, 40%; 18–18.1 min, B, 40%-95%; 18.1–23 min, B, 95%

Mass spectrometry was detected in two modes of ESI+ and ESI-. The samples were separated by UHPLC and analyzed by TOF 5600 mass spectrometer(AB SCIEX). The secondary mass spectrometry was obtained by IDA, which needs to exclude isotopes within 4Da. ESI source conditions, MS/MS conditions and IDA conditions are shown in Table 2. The QC samples were mixed in equal amounts from the samples to be tested, and were tested before, during and after the LC-MS/MS injection. 2 uL of QC was injected, and the QC spacing in the samples was 8 spacings.

**Table 2:** ESI source conditions, MS/MS conditions and IDA conditions.

Project	Condition
Ion Source Gas1(Gas1)	60
Ion Source Gas2(Gas2)	60
Curtain gas (CUR)	30
Source temperature	600°C
IonSapary Voltage Floating (ISVF)	±5500 V (ESI+ mode and ESI- mode)
TOF MS scan m/z range	60–1000 Da
Product ion scan m/z range	25–1000 Da
TOF MS scan accumulation time	0.20 s/spectra
Product ion scan accumulation time	0.05 s/spectra
Declustering potential (DP)	±60 V (both in ESI+ mode and ESI-mode)
Collision Energy	35 ± 15 eV
Candidate ions to monitor per cycle in IDA	6

#### Data processing and statistical methods for untargeted metabolomics

The data obtained from UPLC-Q-TOF-MS were converted into mzXML format by ProteoWizard (Version 3.0, Palo Alto, CA, USA), then the XCMS (https://xcmsonline.scripps.edu/landing_page.php?pgcontent=mainPage) was used for peak area identification, alignment, extraction, peak baseline correction, peak area normalization processing and retention time correction, and the derived ion characteristic fronts were stored in matrix form. Subsequently, the venom group and the control group of sample data were imported into SIMCA-P for multivariate statistical analysis, the data of each group were first examined in general by the PCA method to obtain the general aggregation and separation trends. To further highlight the differences between the two groups and improve the analytical power and validity of the model, the data were analyzed using partial least squares discriminant analysis (PLS-DA). To remove the noise irrelevant to the classification information, maximize the differences between different groups within the model, and improve the analytical ability and validity of the model. We use Orthogonal Partial Least Squares Discriminant Analysis (OPLS-DA). In addition, volcano plots were drawn using R language for unidimensional statistical analysis. *P* < 0.05 was selected as a significant difference in all statistical analyses.

#### Identification of differential metabolites and metabolic pathway analysis

The OPLS-DA model was used to calculate the *VIP*(Variable importance in the projection) values of each metabolite in this model and unidimensional statistical analysis, and metabolites meeting *VIP>1* and *P<0*.*05* were selected as those with significant differences. In addition, the information on differential metabolites were subjected to hierarchical clustering analysis and correlation analysis to evaluate the rationality of differential metabolites and the correlation between metabolites, then the information on differential metabolites was imported into the KEGG database for enrichment analysis of metabolic pathways.

### Targeted metabolomics analysis

#### Preparation of standard solutions and calculation of metabolite content

According to the results of untargeted metabolomics, using a pre-cooled methanol-acetonitrile solution (V _methanol_/V _acetonitrile_ = 1:1) to dilute the concentration of the obtained standard product of differential metabolites to 100 μg/ml to obtain the standard product stock solution. Set 1.5 times the group spacing to dilute the mixed standards to nine concentrations of 2.25, 1.5, 1, 0.675, 0.45, 0.3, 0.2, 0.13, 0.08 μg/ml and wait for injection. After that the standard curves of standard concentration and peak area were plotted with the concentration as the horizontal coordinate and the peak area as the vertical coordinate. Finally, the obtained peak area is substituted into the formula of the standard curve to exactly calculate the content of metabolites.

#### Chromatographic and mass spectrometry conditions for targeted metabolomics

Targeted metabolomics was processed using an ACQUITY UHPLC BEH C18 column(column temperature 40°C, flow rate 0.2 ml/min, injection volume 5 μl). Gradient elution program: mobile phase A (0–3 min, 95%; 3–6 min, A 95% - 30%, 6–7 min, 30%, 7–7.5 min, 30% - 95%,7.5–12 min, 95%). ESI mode was selected according to the difference in untargeted metabolomics.

### Clinical validation of potential metabolic markers for *Naja atra* bite

Based on the results of targeted metabolomics analysis of serum samples from *Naja atra* bitten pigs, key metabolites were selected for targeted quantitative analysis of eight clinical samples. Chromatographic and mass spectrometric conditions, columns, and analytical methods were the same as those for porcine targeted metabolomics. The consistency of metabolites was determined based on retention times and molecular ion peaks of mass spectrometry peaks, and quantitative analysis methods were calculated as above.

### Statistical analysis

All experiments were repeated at least three times, and the results were mean ± standard deviation, statistical analysis and graphing were performed using SPSS 20.0, GraphPad Prism 5 and Origin8. *P < 0*.*05* were considered to indicate significantly different.

## Results

### Establishment of the *Naja atra* bite pig model

#### Calculation of LD_50_ of *Naja atra* venom-injected Mice and *Bama miniature pigs*

The LD_50_ value of 0.803 mg/kg (Table 3) for intramuscular injection of *Naja atra* venom into mice was calculated using the probit model method. Then the theoretical LD_50_ value of 2 mg/kg for intramuscular injection of *Naja atra* venom into Guangxi *Bama miniature pigs* was obtained by the body surface area conversion method.

**Table 3:** Death of mice within 7 days of intramuscular injection of *Naja atra* venom.

Dose/(mg/kg)	logarithmic measurement/X	Number of animals	Number of deaths	Death percentage /%	Experimental probability/Y	Regression probability /Y
1.00	0	10	10	100	——	6.66
0.90	-0.046	10	8	80	5.84	5.86
0.81	-0.092	10	4	40	4.75	5.06
0.72	-0.143	10	2	20	4.16	4.16
0.65	-0.187	10	1	10	3.72	3.39

*Y (Probit) = 6.6559+17.466Log(D)

#### Behavioral observation and wound changes of poisoned pigs

**Fig 1:**
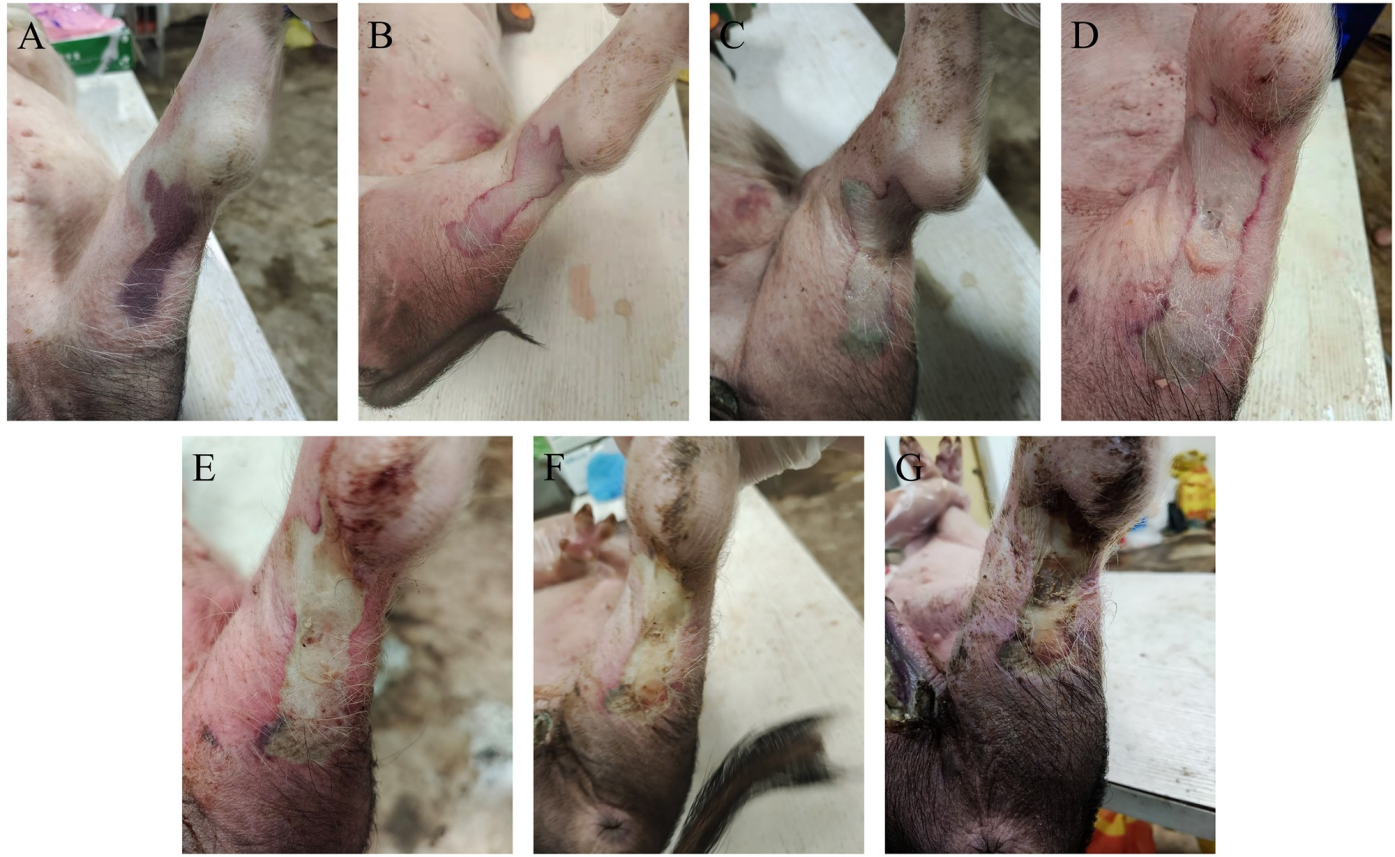
Wound changes of pigs after injection of *Naja atra* venom. With the continuous action of snake venom, (A) bleeding symptoms appear at the wound within 30 minutes, (B) the wound starts to turn white and swollen after 3 hours, (C) exudate appears in the wound after 6 hours, (D) the wound edges are depressed inward with clear edges after 12 hours, (E) a part of crusting appears at the edges after 24 hours, (F) further depression appears in the wound after 48h, (G) the wound is highly crusted after 72 hours and the crusts fall off at a later time.

Compared with the control group, the pigs in the venom group began to show signs of neurotoxin poisoning such as drowsiness, general weakness, drooping eyelids and dilated pupils about 30 min after the injection of snake venom, there also will be a large area of bleeding from the wound of the pig at the same time (Fig 1A). At about 3h (Fig 1B), the injection site began to be white and swollen, the pig was convulsed, and it was difficult to move. After 6 h (Fig 1C), the pig began to lie down and there was exudate from the wound, and after 12 hours (Fig 1D), the tissue at the wound is sunken inward, with clear edges, and scabs falling off in the ensuing time.

#### The results of hematological related indexes analysis of pigs

We put the results of the blood test in Table 4. When the body is poisoned, the number of white blood cells (WBC) will increase. After intramuscular injection of *Naja atra* venom, the WBC of pigs increased within 6 hours, and continued to increase within 12 hours, suggesting that the pigs had a poisoning reaction in 6 hours. Serum enzymatic test results can reflect the effect of venom on the liver, and the continuous increase of glutamic pyruvic transaminase (ALT) and glutamic oxaloacetic transaminase (AST) indicates that the pigs have liver damage during poisoning. The detection of electrolytes is mainly to reflect the functional changes of the pig heart. Creatine kinase-MB (CK-MB) increased continuously within 6 hours and 12 hours, suggesting that the heart of the pigs was attacked by the venom and continued to aggravate after poisoning. Changes in lactate dehydrogenase (LDH) reflect the level of tissue damage, the elevation of LDH reflects that the pigs did experience tissue damage caused by the poisoning. The liver and heart were affected by the venom. At the same time, when acute inflammation occurs or when the body is severely traumatized, hypersensitive C-reactive protein (HS-CRP) will increase rapidly within a few hours, which is highly consistent with our examination results.

**Table 4:** Blood test results before and after *Naja atra* injection.

Blood indexes	Before saline/venom injection		6h after injection of saline/venom		12h after injection of saline/venom	
	Control group	Venom group	Control group	Venom group	Control group	Venom group
WBC(1⊆109 /L)	26.12±0.65	28.37±2.50	24.68±0.88	29.39±1.53*	26.89±2.41	33.50±1.50*
HS-CRP (mg/L)	0.34±0.05	0.30±0.07	0.26±0.07	0.73±0.04**	0.53±0.15	1.01±0.19*
LDH(U/L)	557.67±21.38	580.0±69.47	546.33±13.01	1552±400.6*	505.33±73.93	1913.3±669.4*
AST/ALT	0.80±0.09	0.61±0.12	0.75±0.09	4.13±0.9**	0.63±0.57	3.67±0.67**
CK-MB(U/L)	262.33±44.63	300.0±64.5	256.67±53.63	602.67±193*	252.67±7.63	1119.0±312.1**

* p<0.05

** p<0.01

#### Hematoxylin-eosin staining results

**Fig 2:**
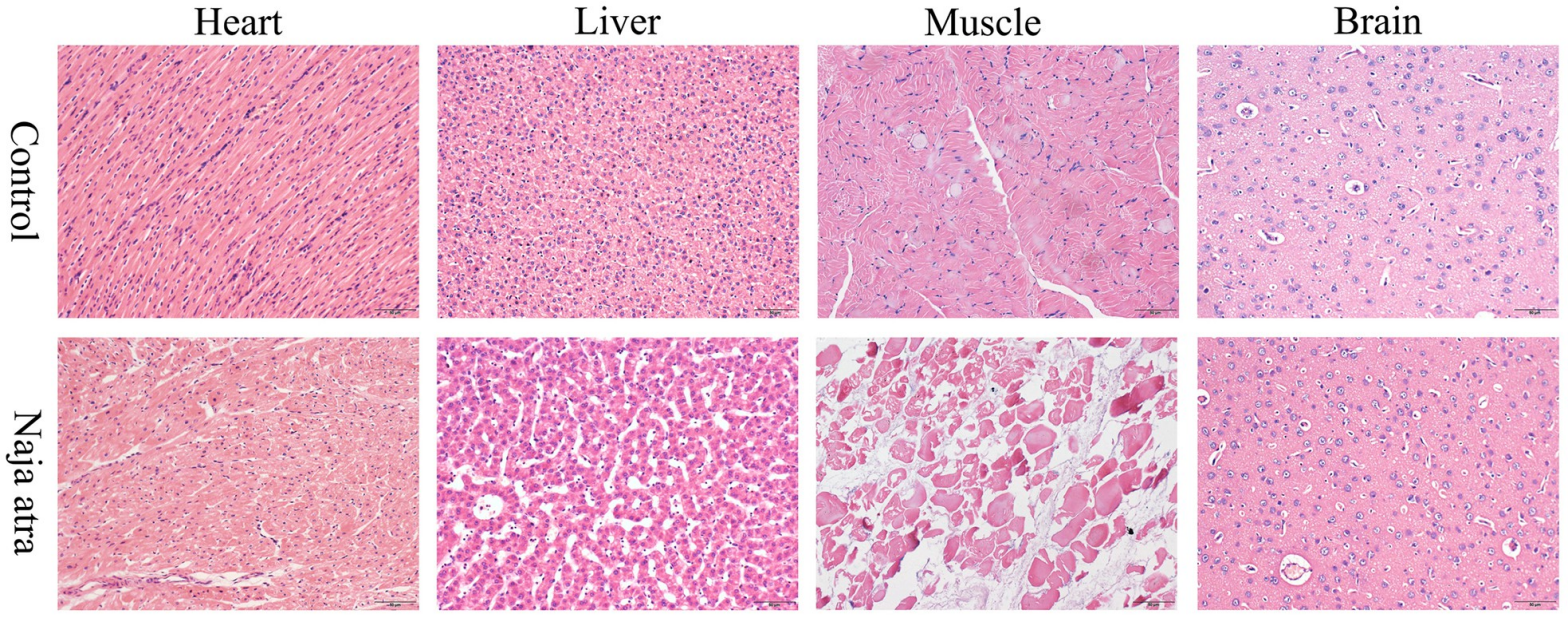
Pathological changes of muscle, liver, heart and brain before and 12 h after injection of *Naja atra* venom. **(×200)** Compared to the control group, the myocardial tissues of the pigs in the snake venom group were disorganized. Large tissue gaps appeared in the liver tissue. Nuclei in muscle tissues were lysed and cytoplasm was arranged extremely irregularly. Swelling of some nerve cells could be observed in the brain tissue.

Compared with the control group, the muscle histopathological findings of the pigs in the venom group showed nuclei lysis and cytoplasm showing irregular arrangement. Myocardial tissues were disorganized, and myocardial cells were wrinkled with different degrees of deformation and necrosis. The liver tissue could be observed to have larger tissue gaps, suggesting that the liver might have hemorrhage and necrosis due to the action of venom, and the structure of liver lobules was lost, with a tendency of cell nuclei lysis. Swelling of some nerve cells could be observed in brain tissue. The results of HE staining are shown in Fig 2.

In conclusion, an animal model of *Naja atra* bite in *Bama miniature pigs* can be successfully constructed at 6h after intramuscular injection of a certain dose (2 mg/kg) of *Naja atra* venom into the legs of pigs.

### Results of serum untargeted metabolomics analysis

#### Quality control and total ion flow plots for serum metabolomics analysis

**Fig 3:**
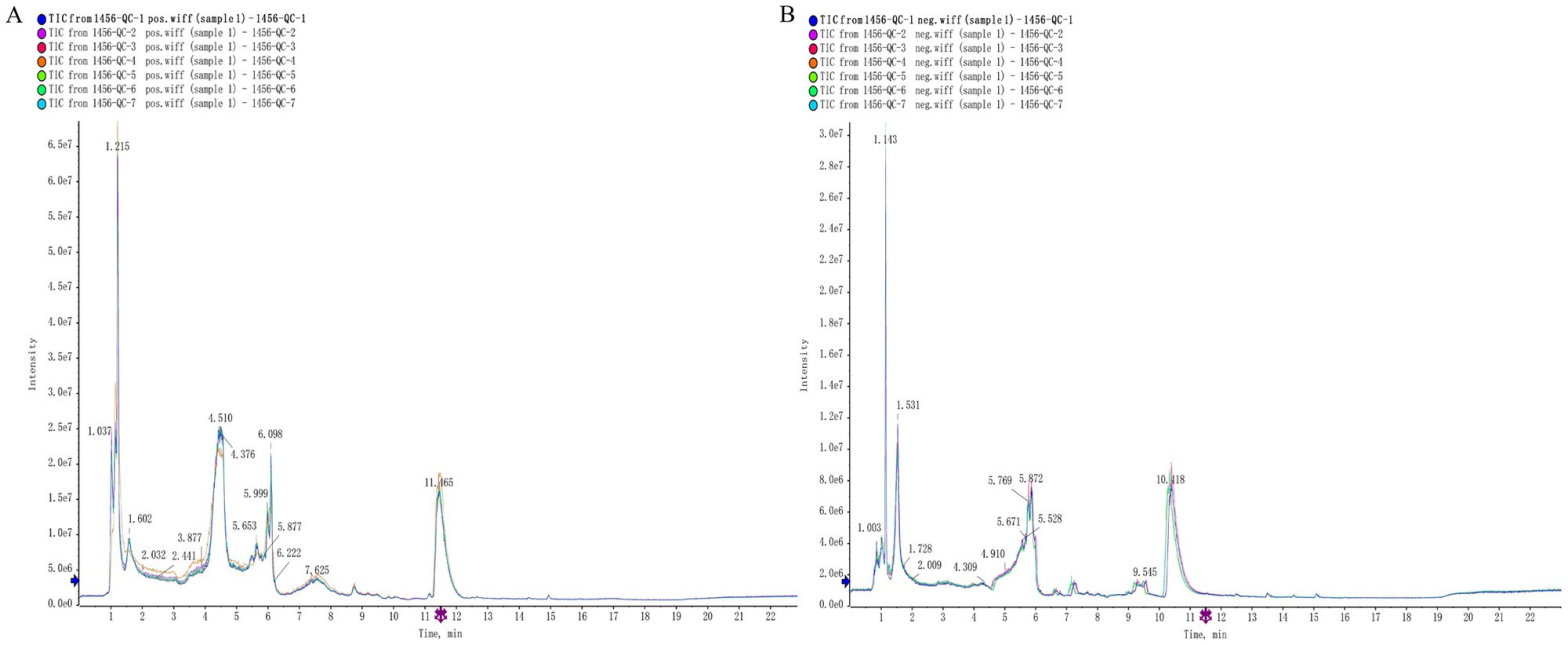
TIC plot of QC samples overlapped in (A) ESI+ and (B) ESI- modes.

In order to ensure the normal use of the instrument, we compared the total ion chromatograms (TIC) of the QC samples in ESI+ and ESI- (Fig 3). It shows that the machine operated well during the use and the variation caused by the instrument error was small throughout the experiment. and the response intensity of each chromatographic peak basically overlapped with the retention time. Then, the serum samples were detected in ESI+ and ESI-, the metabolites in the samples were collected by the UPLC-Q-TOF-MS system, and the metabolite ion peaks were extracted by XCMS software (S1). 5045 chromatographic peaks were identified in positive ion mode and 3871 chromatographic peaks were identified in negative ion mode. These ion features will be stored in matrix form and used for subsequent multivariate analysis model establishment.

#### Multidimensional statistical analysis

**Fig 4:**
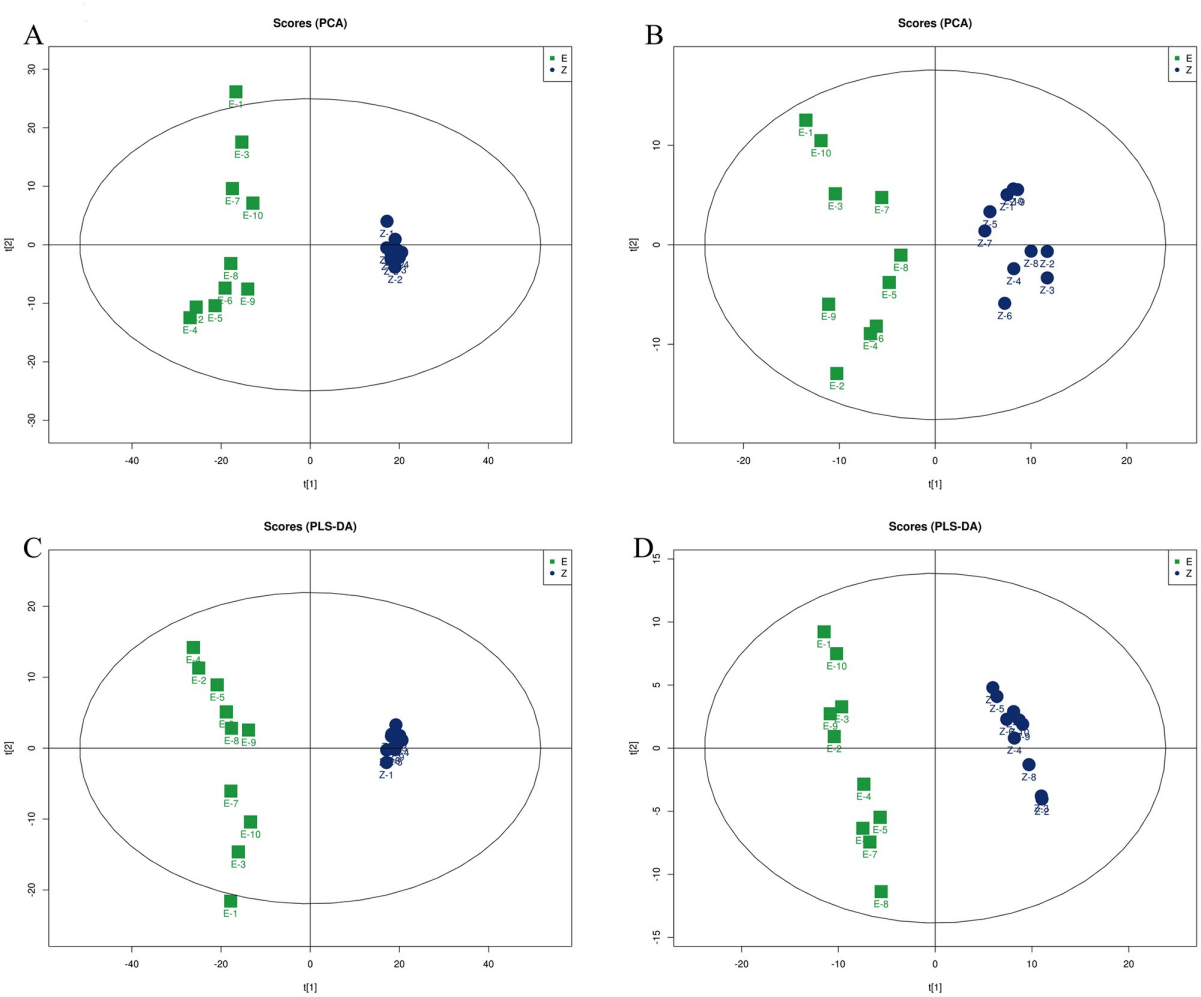
Diagnostic model based on data of untargeted metabolomics. **PCA score plots of serum metabolic profiling of the Venom group and Control group (A) ESI+, (B)ESI-**. In the PCA model, the score parameter (R^2^X) was 0.73 in ESI+ and 0.511 in ESI-. **PLS-DA score plots of serum metabolic profiling of the Venom group and Control group (C) ESI+, (D) ESI-.** In the PLS-DA model, the score parameters R^2^X is 0.724, R^2^Y is 0.985, and Q^2^ is 0.971 in ESI+. the score parameters R^2^X is 0.324, R^2^Y is 0.991, and Q^2^ is 0.932 in ESI-.

**Fig 5:**
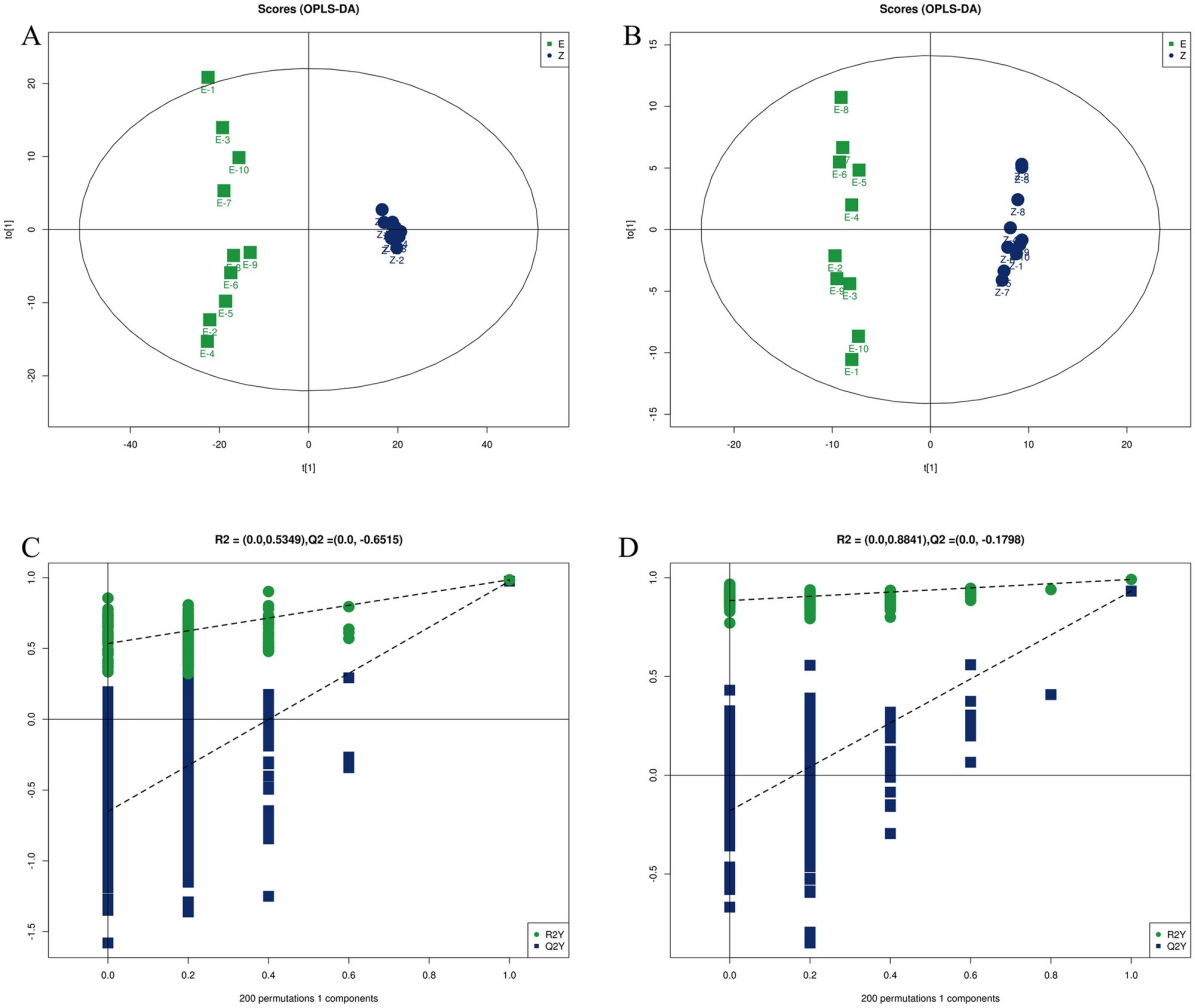
Diagnostic model based on data of untargeted metabolomics. **OPLS-DA score plots of serum metabolic profiling of the Venom group (E) and Control group(Z). (A)** ESI+, (B)ESI-. **Permutation plots of the OPLS-DA models (C) ESI+, (D)ESI-.** In the OPLS-DA model, the model parameters obtained from the seven-cycle interaction validation were as follows, with score parameters R2X of 0.724, R2Y of 0.985, and Q2 of 0.975 in ESI+. score parameters R2X of 0.324, R2Y of 0.991, and Q2 of 0.932 in ESI-. After building the OPLS-DA model 200 times to obtain the characteristic parameters of the stochastic model, the characteristic parameters R2 was 0.5349 and Q2 was -0.6515 in ESI+ and R2 was 0.08841 and Q2 was -0.1798 in ESI-.

The obtained data were normalized by Pareto-scaling, and Principal Component Analysis (PCA) (Fig 4A and 4B) was performed between the control group and the venom group. In this model, R^2^X reflects the degree of interpretation of the established model to the variable. The closer R^2^X is to 1, the higher the degree of interpretation of the variable by the established model, the score reflects the variability between the two groups and the data within each group variability. The green dots represent the experimental group (E) and the blue dots represent the control group(Z) of samples. The ESI+ score parameter (R^2^X) was 0.73, and the ESI- score parameter (R^2^X) was 0.511. The results showed that the data were cleanly separated between the control and venom groups, the clustering between the groups was better, the PCA model could effectively separate the two, and the differences between the two groups could be explained to a great extent by the first principal component (PC1). The data were analyzed using Partial Least Squares Discriminant Analysis (PLS-DA) (Fig 4C and 4D). In this model, R^2^X and R^2^Y denote the explanation rate of the proposed model for the X and Y matrices, respectively, and Q^2^ labels the predictive power of the model. We found that the comparison between the control group and the venom group were R^2^X = 0.724, R^2^Y = 0.985, Q^2^ = 0.971 in ESI+ mode, and R^2^X = 0.324, R^2^Y = 0.991, Q^2^ = 0.932 in ESI- mode. The score parameters of the PLS-DA model further indicate that our model is stable and reliable. At the same time, we found that the Q^2^ between the two groups is greater than 0.5, indicating that the established PLS-DA model can well explain and predict the difference between the two groups of samples. We used orthogonal partial least squares discriminant analysis (OPLS-DA) to analyze the data between the control group and venom groups (Fig 5A and 5B). The model evaluation parameters obtained by 7-cycle interactive verification are R^2^X = 0.724, R^2^Y = 0.985, Q^2^ = 0.975 in ESI+ mode, and R^2^X = 0.324, R^2^Y = 0.991, Q^2^ = 0.932 in ESI- mode. These data further demonstrate that our constructed model is stable and data reliable. At the same time, a response permutation testing (PRT) experiment was performed on the constructed OPLS-DA model. By randomly changing the arrangement order of the categorical variable Y, the OPLS-DA model was established 200 times to obtain the R^2^ and Q^2^ values of the random model. The R^2^ intercept is 0.534 and the Q^2^ intercept is -0.651 in ESI+ mode, and the R^2^ intercept is 0.0884 and the Q^2^ intercept is -0.179 in ESI- mode (Fig 5C and 5D). To sum up, the models constructed by the three models can be distinguished well and the separation trend is obvious, and they have good predictive ability and reliability in both the ESI+ model and the ESI- model.

#### Univariate statistical analysis of serum metabolites

**Fig 6:**
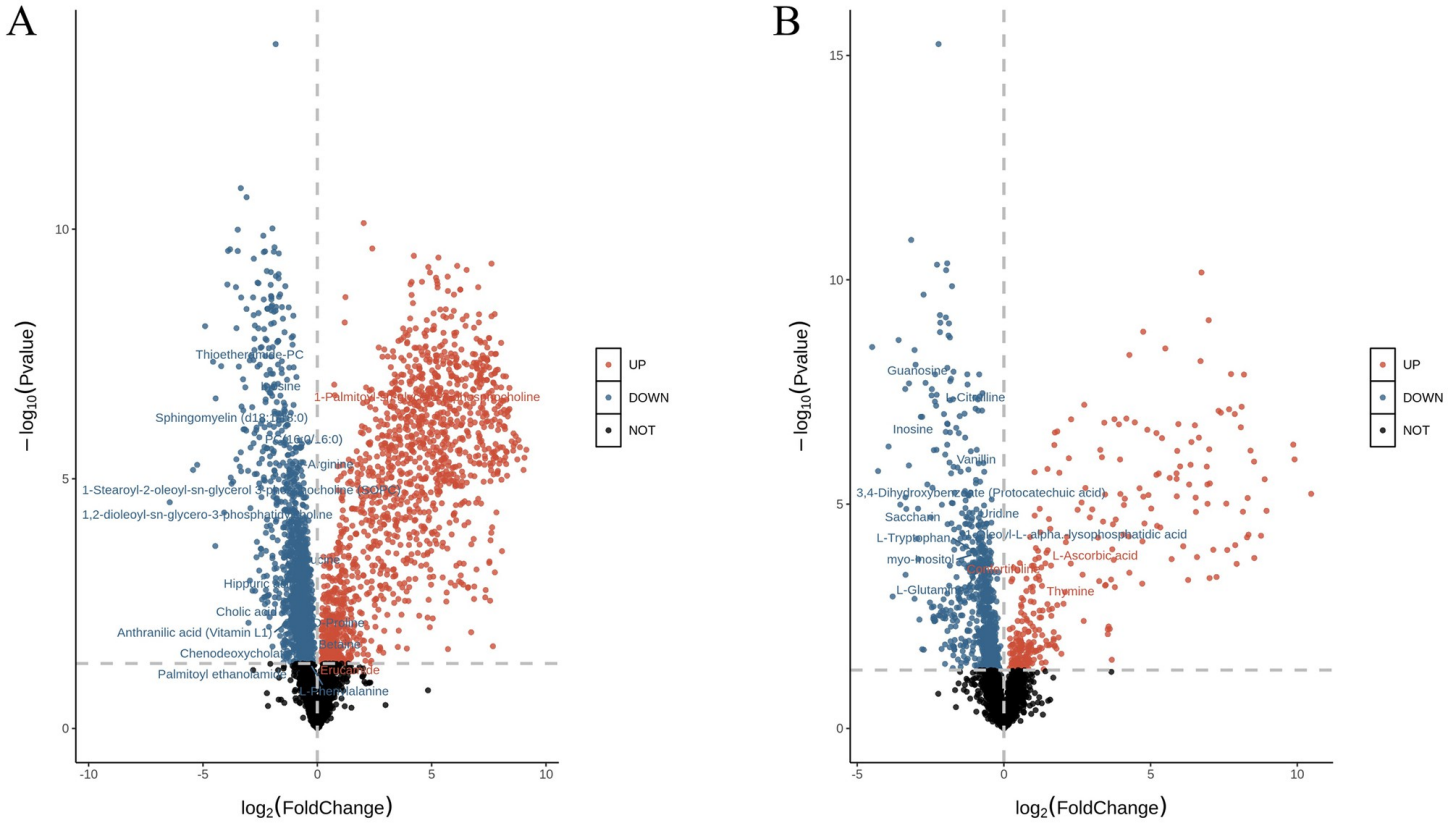
Volcano plot of positive and negative ion mode data. **A: ESI+, B: ESI-.** Metabolites with adjusted p-value < 0.05 and log2FoldChange > 1 will be displayed as red dots, metabolites with adjusted p-value < 0.05 and log2FoldChange < -1 will be displayed as blue dots, and metabolites with adjusted p-value > 0.05 will be displayed as black dots, and the dispersion of red and blue dots indicates a difference between metabolites in the venom group and the control group.

Volcano plots were made using univariate analysis to show the significance of metabolite changes between the two samples (Fig 6A and 6B). The red dots and blue dots are metabolites with *FC > 1*.*5* and *P < 0*.*05*, with a scatter distribution. The further away from the origin, the greater the contribution of the metabolite to the difference between groups, the greater the difference between the control group and the venom group. From our results, the metabolites of the venom group and the control group are significantly different in the ESI+ and ESI-.

#### Screening and identification of differential metabolites

The OPLS-DA model was constructed, VIP>1, and P<0.05 were used as screening criteria, and the mass spectra that met the conditions were compared with the corresponding spectra in the Metlin, Human Metabolite Database, Massbank, and KEGG databases to obtain putative metabolites. In ESI+, 22 metabolites with significant differences were screened and identified, 2 metabolites increased in expression, and 20 metabolites decreased in expression. In ESI-, 36 metabolites with significant differences were screened and identified, 7 metabolites increased in expression and 29 metabolites decreased in expression. Inosine, glycocholic acid, hippuric acid, chenodeoxycholate, clycochenodeoxycholate, arginine, leucine, phenylalanine can be identified in ESI+ and ESI- (Table 5).

**Table 5:** Differential metabolites identified in ESI+ and ESI-.

Putative Metabolites	m/z	RT(s)	VIP	p-value	Ion mode	Trend
Thioetheramide-PC	780.55	268.40	2.76	0.000	ESI+	↓
Inosine	269.09	420.13	1.50	0.000	ESI+	↓
1-Palmitoyl-sn-glycerol-3-phosphocholine	518.32	399.55	1.69	0.000	ESI+	↑
Sphingomyelin (d18:1/18:0)	794.60	260.58	2.67	0.000	ESI+	↓
PC(16:0/16:0)	734.57	278.25	2.68	0.000	ESI+	↓
Arginine	175.12	1007.29	1.85	0.000	ESI+	↓
1-Stearoyl-2-oleoyl-sn-glycerol 3-phosphocholine (SOPC)	810.60	112.88	3.14	0.000	ESI+	↓
1,2-dioleoyl-sn-glycerol-3-phosphatidylcholine	786.60	128.01	6.03	0.000	ESI+	↓
Leucine	132.10	527.63	1.37	0.000	ESI+	↓
Hippuric acid	180.06	369.02	1.04	0.002	ESI+	↓
Cholic acid	409.29	409.63	1.59	0.002	ESI+	↓
Glycocholic acid	466.31	468.90	1.09	0.002	ESI+	↓
1-Oleoyl-sn-glycerol-3-phosphocholine	522.35	361.05	3.16	0.003	ESI+	↓
D-Mannose	198.10	596.25	1.75	0.004	ESI+	↓
Glycochenodeoxycholate	450.32	416.66	1.01	0.004	ESI+	↓
Anthranilic acid (Vitamin L1)	138.05	551.85	1.36	0.006	ESI+	↓
Proline	116.07	602.16	1.96	0.007	ESI+	↓
Chenodeoxycholate	410.32	304.46	1.22	0.013	ESI+	↓
Betaine	118.09	578.42	1.02	0.024	ESI+	↓
Erucamide	338.34	67.75	1.40	0.038	ESI+	↑
Phenylalanine	166.09	488.45	1.32	0.046	ESI+	↓
Palmitoyl ethanolamide	300.29	72.73	1.07	0.048	ESI+	↓
Guanosine	282.08	478.16	1.83	0.000	ESI-	↓
Citrulline	174.09	729.00	1.49	0.000	ESI-	↓
Inosine	267.07	403.11	5.42	0.000	ESI-	↓
Vanillin	151.04	70.20	4.63	0.000	ESI-	↓
3,4-Dihydroxybenzoate (Protocatechuic acid)	153.02	52.88	2.25	0.000	ESI-	↓
Uridine	243.06	288.24	1.19	0.000	ESI-	↓
Saccharin	181.99	61.17	2.79	0.000	ESI-	↓
1-Oleoyl-L-.alpha.-lysophosphatidic acid	457.23	456.82	1.05	0.000	ESI-	↓
Tryptophan	203.08	466.49	1.90	0.000	ESI-	↓
myo-Inositol	179.06	739.25	1.21	0.000	ESI-	↓
Ascorbic acid	197.01	216.78	1.93	0.000	ESI-	↑
Glutamine	145.06	695.01	2.41	0.000	ESI-	↓
Thymine	125.03	185.65	1.27	0.000	ESI-	↑
Confertifoline	233.15	61.48	3.43	0.000	ESI-	↓
Palmitic acid	255.23	97.08	10.95	0.001	ESI-	↓
2-Oxoadipic acid	141.02	620.02	8.19	0.001	ESI-	↓
Leucine	130.09	478.56	4.45	0.002	ESI-	↓
Arachidonic Acid (peroxide free)	303.23	93.02	11.45	0.002	ESI-	↑
4-Pyridoxic acid	182.05	88.31	1.43	0.002	ESI-	↓
Hippuric acid	178.05	366.52	2.96	0.003	ESI-	↓
Glycocholic acid	464.30	460.56	2.92	0.003	ESI-	↓
Valine	116.07	555.48	1.58	0.005	ESI-	↓
Caffeic Acid	179.03	190.79	1.12	0.006	ESI-	↓
Glycochenodeoxycholate	448.31	393.99	1.63	0.009	ESI-	↓
Arginine	173.10	967.47	1.33	0.011	ESI-	↓
Thymidine	241.08	186.28	3.10	0.013	ESI-	↑
Chenodeoxycholate	451.31	285.77	3.09	0.014	ESI-	↓
2-Ethyl-2-Hydroxybutyric acid	131.07	258.67	1.94	0.015	ESI-	↓
DL-lactate	89.02	571.16	1.07	0.016	ESI-	↓
DL-3-Phenyllactic acid	165.06	194.50	4.18	0.017	ESI-	↑
L-Gulonic gamma-lactone	177.04	264.81	2.39	0.017	ESI-	↑
ketoisocaproic acid	129.06	97.55	5.73	0.027	ESI-	↓
Alpha-D-Glucose	179.06	558.06	2.61	0.031	ESI-	↓
Glutamate	146.05	751.20	1.20	0.033	ESI-	↓
m-Chlorohippuric acid	213.02	176.44	1.25	0.043	ESI-	↑
Phenylalanine	164.07	466.42	2.13	0.083	ESI-	↓

*↑indicates that the content is increased, ↓indicates that the content is decreased

#### Cluster analysis and correlation analysis of differential metabolites

**Fig 7:**
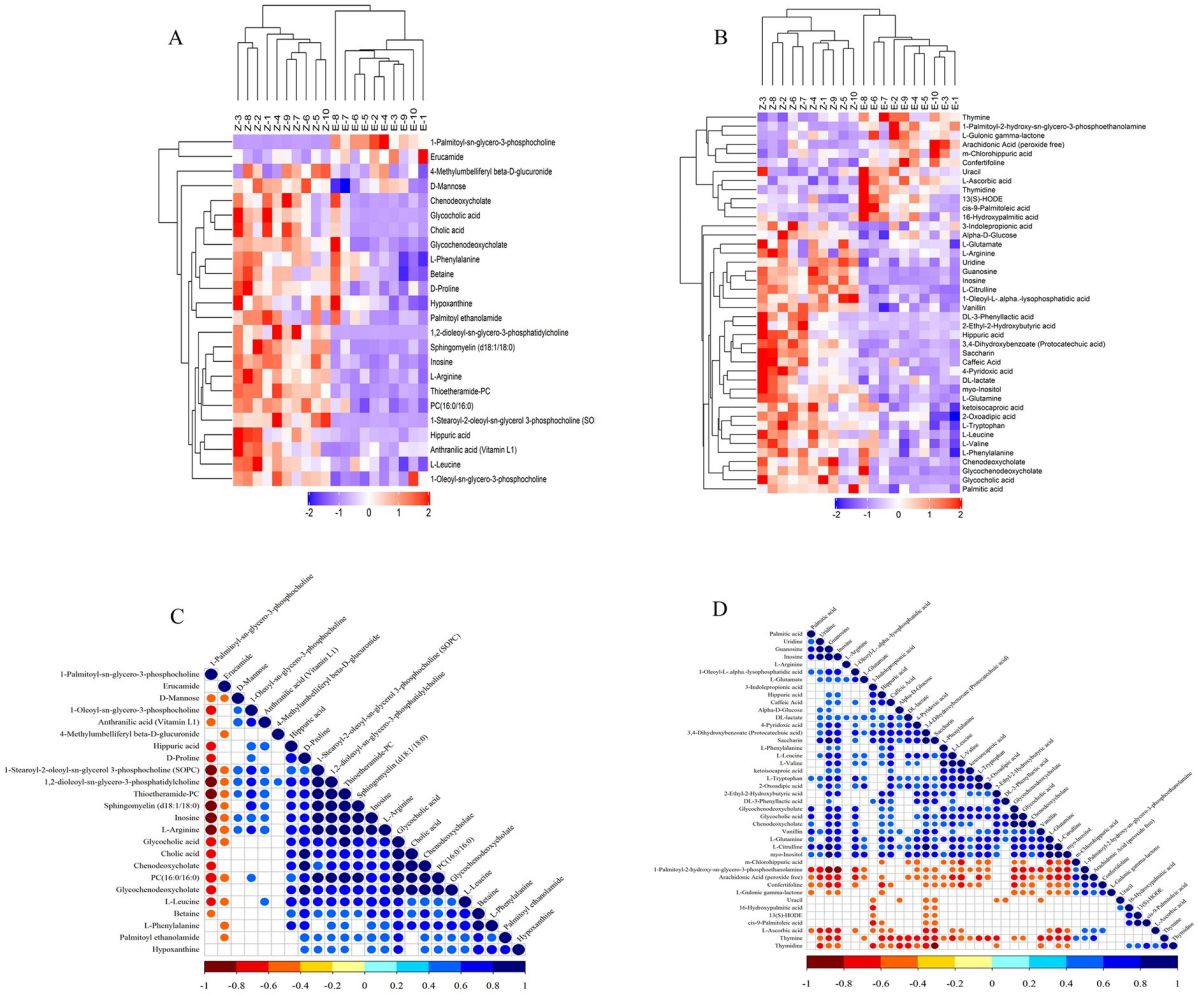
**Hierarchical clustering results of significant differences in ESI+(A) and ESI-(B)**. Hierarchical cluster analysis reflects the fact that candidate differential metabolites have similar properties or characteristics in some aspects. The fact that different metabolites are classified into the same cluster indicates that these metabolites are likely to be affected at the same time because of the toxic conditions. **Correlation analysis results of significant differences in ESI+ (C) and ESI- (D).** The purpose of correlation analysis is to see the consistency of metabolite and metabolite trends, and to analyze the correlation between individual metabolites by calculating the Pearson correlation coefficient between two pairs of all metabolites. Metabolite correlations often reveal the synergistic nature of changes between metabolites, for example, the same trend of change of a certain type of metabolite is a positive correlation, indicated as a red dot, and the opposite trend of change with a certain type of metabolite is a negative correlation, indicated as a blue dot, while the darker the color indicates a higher correlation.

In order to evaluate the rationality of the candidate metabolites, the 58 differential metabolites identified were analyzed for biological functions. The expression level of the differential metabolites was used to perform hierarchical cluster analysis (Fig 7A and 7B) for each group of samples, In the results of hierarchical cluster analysis, different metabolites are grouped into the same cluster, which indicates that these metabolites have similar properties or characteristics in some aspects, for example, they may be involved in similar metabolic pathways or reactions, or they may be affected simultaneously under certain physiological or pathological conditions. It can be seen that the same group of samples can appear in the same cluster by clustering, indicating that the screened differential metabolites are reasonable and accurate. To further understand the correlation between the significant differential metabolites in the process of biological change, correlation analysis (Fig 7C and 7D) was performed on the differential metabolites, the differential metabolites had a strong correlation.

#### KEGG pathway enrichment analysis of differential metabolites in pig serum

**Fig 8:**
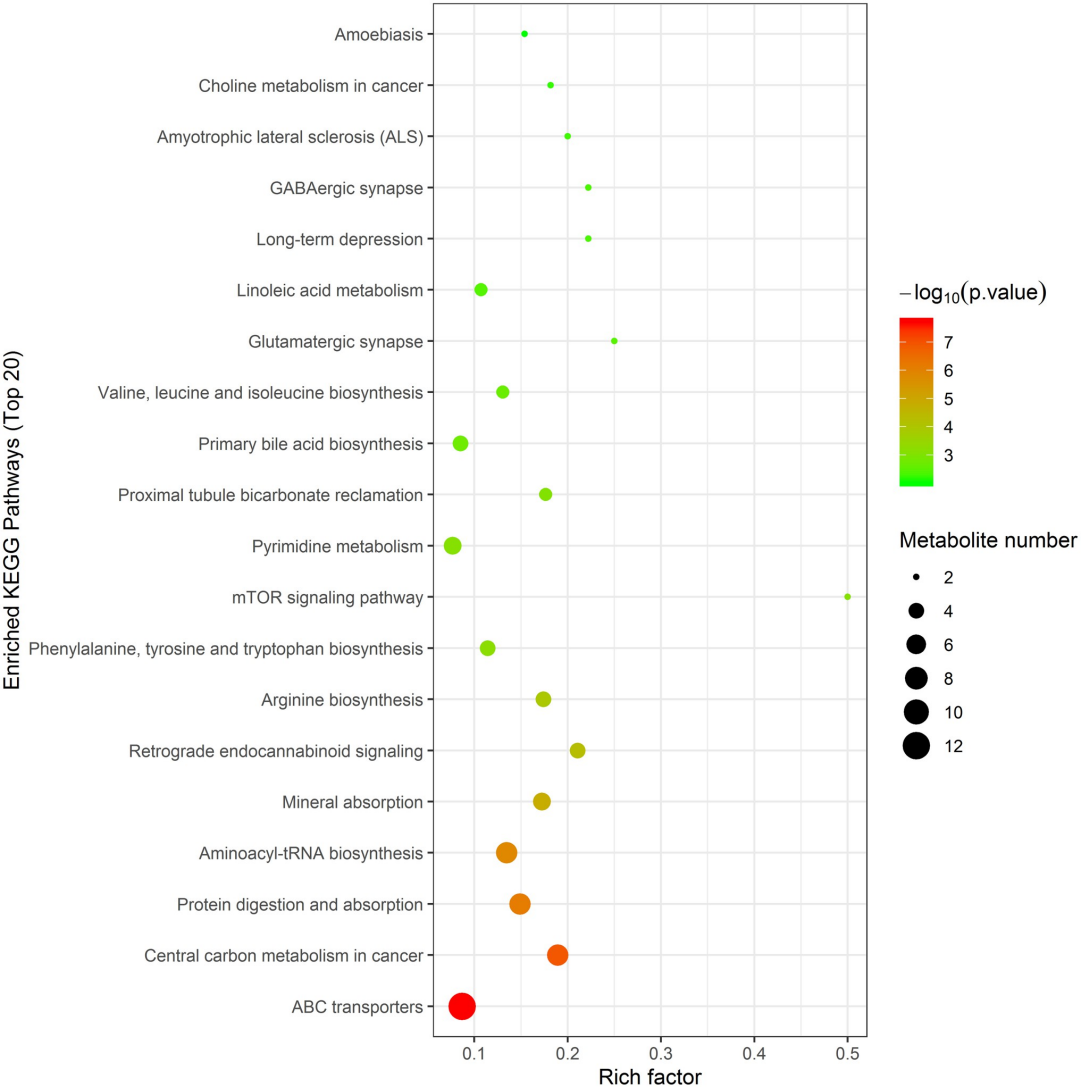
Enrichment analysis of differential metabolite KEGG pathway. The x axis indicates the rich factor corresponding to each pathway, and the y axis indicates the name of the KEGG metabolic pathway. The size and color of bubbles represent the number and degree of enrichment of different metabolites, respectively.

KEGG pathway enrichment analysis was performed on the identified differential metabolites by Fisher’s exact test. Fig 8 lists 20 metabolic pathways associated with *Naja atra*-bited pigs, and 12 metabolites appear in ABC transporters, with 8 appearing in Central carbon metabolism in cancer.

### Targeted metabolomic analysis of differential metabolites

**Fig 9:**
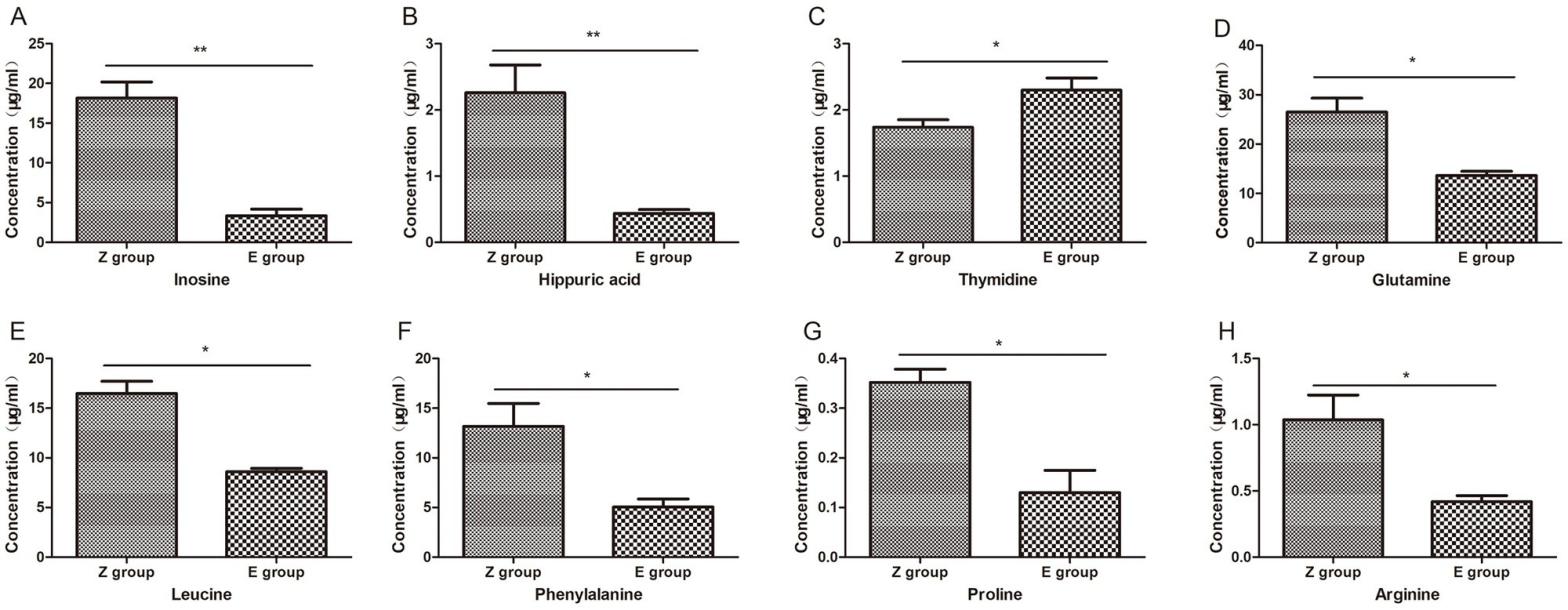
Targeted metabolomics quantitative calculation results of differential metabolites. Z group(control group), E group(venom group). Compared with the control group, glutamine, leucine, proline, arginine, phenylalanine, inosine, and hippuric acid levels were decreased and thymidine was increased in the venom group, and the trends were consistent with the changes in non-targeted metabolomics results. (* p<0.05, ** p<0.01).

According to the different chromatographic peak areas generated by different concentrations of standard products, the corresponding standard curves are drawn. The parameter R^2^ values of the curves are all greater than 0.99, The standard curve can be used for quantitative calculation. Leucine, proline, arginine, phenylalanine and chenodeoxycholic acid were validated in ESI+ mode. glutamine, inosine, hippuric acid and thymine were validated in ESI-mode (S2). The retention times of metabolites in the control group (Z group) and venom group (E group) are the same as those of the standard. Compared with the control group, the serum contents of glutamine, leucine, proline, arginine, phenylalanine, inosine, and hippuric acid in the venom group decreased, while the thymidine increased, and their changing trends same as untargeted metabolomics results changed consistent (Fig 9). The difference in Chenodeoxycholate between the two groups was not statistically significant.

### Clinical validation of potential metabolic markers for *Naja atra* bite

**Fig 10:**
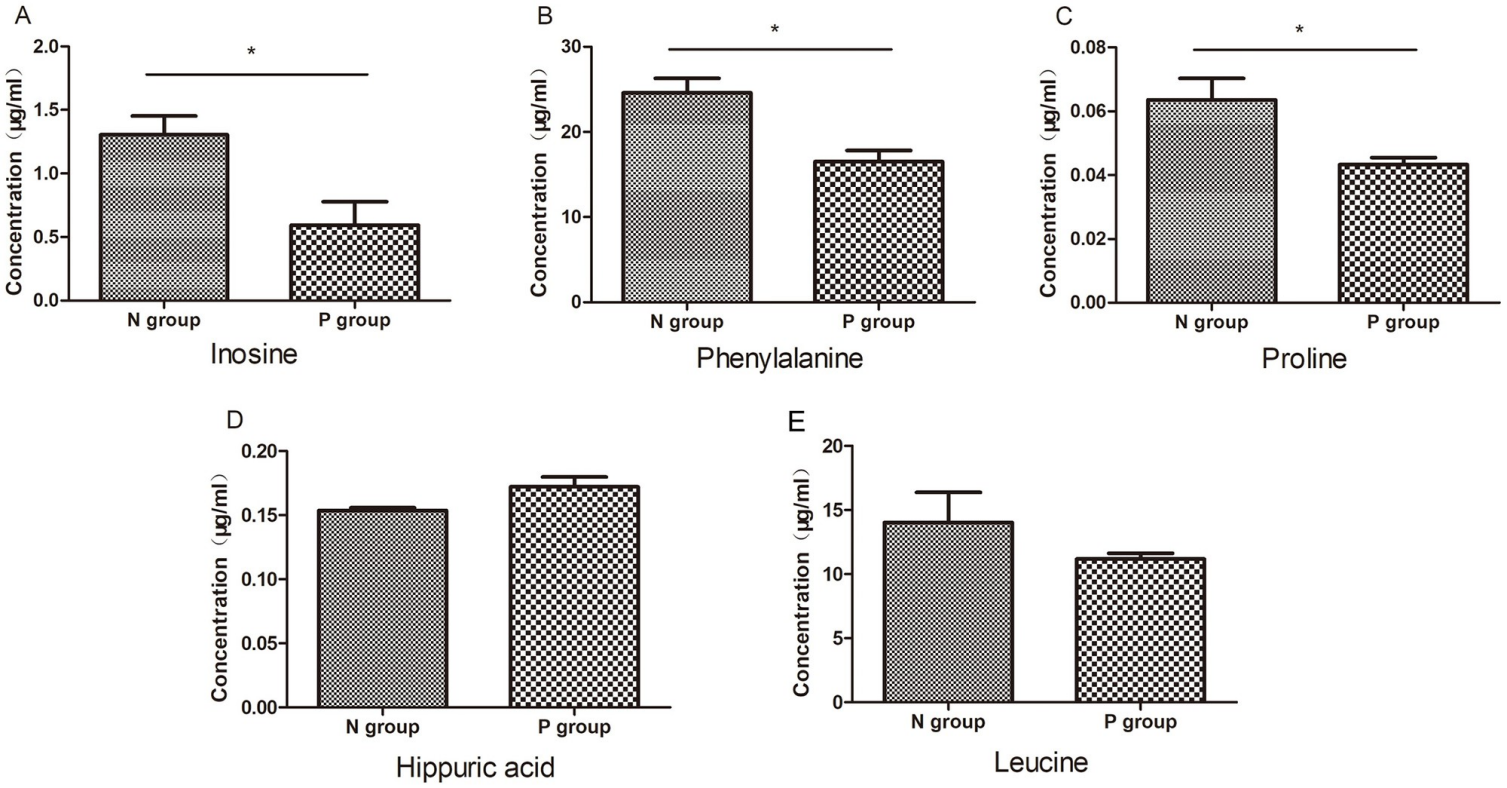
Targeted metabolomics quantitative calculation results of differential metabolites in clinical patients. N group (normal group), P group (patient group). (A) Inosine (B) Phenylalanine (C)Proline (D) Hippuric acid (E) Leucine. Compared with the normal group serum, three metabolite levels of proline, inosine and phenylalanine were reduced in the patient group with statistically significant differences, and there were no differences in the changes of mareic acid and leucine. (* p<0.05).

In order to further verify the reliability of the potential metabolic markers, the above eight metabolites were compared between the normal group (N group) and the *Naja atra* bite patient group (P group), and the absolute quantification of these eight metabolites was performed. We identified leucine, proline, phenylalanine, inosine, hippuric acid in the patient’s serum. Compared with the serum of the normal group, the levels of three metabolites of proline, inosine and phenylalanine in the patient group were decreased, and the difference was statistically significant (Fig 10). In addition, the retention times of glutamine, arginine and thymidine in the control group and the patient group are inconsistent, so the peak area cannot be calculated in this test according to their retention time, and the changes of leucine and hippuric acid did not have statistical significance. These results indicated that proline, inosine and phenylalanine were closely related to the changes in body physiology and pathology after the *Naja atra* bite.

## Discussion

*Naja atra* bite are a worldwide problem, especially in tropical and subtropical regions. In China, the number of *Naja atra* bite is the second highest in the incidence of snake bites. *Naja atra* bite usually result in severe local tissue damage or even tissue necrosis, and the pathophysiological mechanisms of their bites are very complex [2]. At the same time, in clinical practice, the description of the patient’s bite animal upon admission influences the clinician’s medication, and a timely and rapid diagnosis of whether a particular type of venomous snake bite is present is obviously of great help to the physician’s medication, so the search for biomarkers of multiple snake bites has become a high priority [19,20].

In our study, we established a model of *Naja atra* bite in pigs to overcome the limitations of using rats, mice or rabbits as animal models of snakebite in the past. These small animals differ significantly from humans in terms of genetics, body size and lifespan, whereas *Bama miniature pigs* are morphologically homogeneous, genetically stable and well tolerated, and are well suited as model animals for *Naja atra* bite models [21]. However, the number of clinical samples and the sensitivity of metabolomics techniques make the experiment somewhat limited. After the successful establishment of the model, we used untargeted metabolomics to compare the changes of metabolites in the serum of normal and model pigs, and then used targeted metabolomics to absolutely quantify the content of the differential metabolites after finding the appropriate differential metabolites. Eventually, we found that proline, phenylalanine, and inosine are expected to be key metabolites in *Naja atra* bite.

### Relationship between proline, phenylalanine, inosine and *Naja atra* bite

*Naja atra* venom is a protein mixture primarily composed of three-finger toxin, phospholipase A2, and metalloproteinases [22–25]. The sustained action of toxic proteins leads to both local tissue necrosis and systemic neurotoxicity [26]. Snake venom metalloproteinase primarily acts on type IV collagen and certain coagulation factors, causing collagen degradation, inducing apoptosis, and inhibiting platelet aggregation leading to bleeding [27]. When the basement membrane of the skin barrier is breached, phospholipase A2 binds to various receptors in the plasma and axonal membranes of myocytes, causing acute skeletal muscle necrosis, local tissue inflammation with edema and the painful influx of leukocytes into the wound [25,28,29]. The pathophysiological changes we observed in pigs are consistent with the performance of these studies. A study reported that proline plays a great role as a non-essential amino acid in the process of wound healing [30]. The essence of wound healing is the formation of scar tissue, which is mainly composed of collagen, which means that the synthesis, breakdown and resynthesis of collagen are important factors in rebuilding tissue integrity [31]. From a metabolic point of view, the reconstruction of collagen at the wound requires a large number of amino acids and ATP, of which proline is one of the important substrates. Enrichment analysis revealed that proline is involved in arginine and proline metabolism along with arginine, and that the body stores enough arginine to maintain normal muscle and connective tissue mass, but not enough to repair collagen synthesis after injury and accelerate injury healing, and that arginine in the body decreases substantially under severe stress and that endogenous arginine synthesis is insufficient to meet the demand for increased protein turnover. Therefore, arginine is necessary for wound healing and maintenance of positive nitrogen balance [32]. This suggests to us the possibility that arginine and proline metabolism may play an important role in the pathophysiological process of snake bites. Consequently, the decrease in serum proline levels reflects the body’s depletion of this amino acid to repair tissue damage caused by *Naja atra* bite. This reduction is crucial for reconstructing the skin tissue barrier following the bite. In addition, our study also revealed decreased phenylalanine levels in patients bitten by *Naja atra*. Phenylalanine, as an essential amino acid, can only be taken up from food and is transported intracellularly by ABC transporters, and is taken up as a precursor to monoamine neurotransmitters such as dopamine, norepinephrine, and epinephrine. The results of enrichment analysis showed that phenylalanine is also involved in Central carbon metabolism in cancer, Mineral absorption, Phenylalanine metabolism, Phenylalanine, tyrosine and tryptophan biosynthesis [33]. This suggests that these pathways play an important role in snake bites. Studies have shown that neurotoxins can bind to nicotinic and muscarinic acetylcholine receptors, causing symptoms such as peripheral respiratory paralysis [34,35], that phenylalanine as a substrate for the synthesis of monoamine neurotransmitters can strengthen the excitation of the central nervous system, and that phenylalanine depletion in the body may be used to maintain the excitation of the central nervous system, while insufficient intake may also lead to lower serum levels of Phenylalanine level was reduced [36]. Cytotoxins, a member of the three-finger family of toxins, are structurally conserved but possess a wide range of biological activities [37]. Cytotoxins cause depolarization of excitatory tissues (skeletal muscle, cardiac muscle and neural tissue), and studies have reported that cytotoxin binding to cardiac muscle cells leads to cardiomyocyte lysis and ultimately to heart failure [38]. Similarly, depolarization of skeletal muscle can lead to impairment of the motor system. Inosine, an essential metabolite for purine synthesis and degradation, is also transported into the cell mediated by the ABC transporter [39], and by the results of enrichment analysis we found that the purine metabolism involved in inosine was perturbed after snake venom invasion, and the levels of glutamine and inosine were reduced, on the one hand, we speculate that this could be the result of accelerated purine metabolism, and on the other hand suggest that the perturbation of purine metabolism may play a role in the pathophysiological processes of *Naja atra* bite patients [40]. It has also been found that inosine possesses neuroprotective, cardioprotective and immunomodulatory effects in different experimental models by modulating oxidative stress and inflammatory responses [41–43] and that inosine has therapeutic effects in improving motor function during neurological injury or stroke [44]. The decrease in inosine levels may be a response by the body to neurological, motor and immune system damage brought about by *Naja atra* bite.

### Differences between bites of *Naja atra*, *Bungarus multicinctus*, and *Trimeresurus stejnegeri*

The *Naja atra* and the *Bungarus multicinctus* belong to the *Elapidae*, while the *Trimeresurus stejnegeri* belongs to the *Viperidae* [45]. The composition of the venom is the same for snakes belonging to the same family, but the proportion of each component in the venom varies. The *Naja atra* has 64% of the three-finger toxin, while the *Bungarus multicinctus* has 29%. Phospholipase accounted for 19% of the *Naja atra* venom and 37% of the *Bungarus multicinctus* [46]. *Naja atra* venom also contains 5% of metalloproteinases, but metalloproteinases make up a very low percentage of the venom of *Bungarus multicinctus* venom. The composition of venom varies widely among snakes that do not belong to the same family [47], for example, the *Trimeresurus stejnegeri*, the *Deinagkistrodon*, and so on. The venom of the *Trimeresurus stejnegeri* consists mainly of phospholipase A2, metalloproteinase, L-amino acid oxidase, and C-type lectin [48]. The similarities and differences between these snake venom components and levels result in different bite outcomes. For example, *Naja atra* bite result in severe tissue necrosis and systemic neurotoxicity, while *Bungarus multicinctus* bites are predominantly neurotoxic, with little change at the wound site. The difference is that the primary outcome of *Trimeresurus stejnegeri* bites is massive bleeding and coagulation dysfunction in the wound, with far less impact on the nervous system than *Naja atra* and *Bungarus multicinctus* [49]. From the results of metabolite profiles and enrichment analyses outlined by non-target metabolomics, *Naja atra* and *Bungarus multicinctus*s, which belong to the same cobra family, have significant similarities. For example, their variable metabolites and metabolic pathways partially overlap, with metabolites such as proline, guanine, glutamine, leucine, thymidine, etc. Metabolic pathways include mineral uptake, Central carbon metabolism in cancer, Protein digestion and absorption, Aminoacyl-tRNA biosynthesis, and the ABC transporter pathway. However, among these metabolites, some metabolites are different from those of the silver ring snake bite, such as phenylalanine, inosine, etc. This also suggests to us that these differing metabolites could be signature differences between different snake species of the same family. The metabolomic results showed that the metabolites in the *Viperidae* bites of pigs with *Trimeresurus stejnegeri* were very different from those of pigs with *Naja atra* bite. The key metabolites identified in the *Trimeresurus stejnegeri* bite were deoxycholic acid, lithocholic acid, tryptophan and hypoxanthine, and the key metabolic pathways were Liver disease due to cystic fibrosis, Hyperbaric oxygen exposure, Biliary cirrhosis and Tryptophanuria. Compared with thekey metabolites in *Naja atra*bites, hypoxanthine, arginine and tryptophan were common metabolites and showed the same trend, suggesting some similarities between our *Naja atra* bites and the bites of *Trimeresurus stejnegeri* [17,18]. The changes in these metabolites suggest that the changes in metabolites of *Naja atra* bite, *Bungarus multicinctus* bite and *Trimeresurus stejnegeri* bite pigs are related to the composition of snake venom, and also lay the foundation for the next exploration of the effects of specific components of snake venom on the metabolites and metabolic pathways of the body.

Our established model of *Naja atra*bite in large animals has some reference and research value, and the potential biomarkers we explored can help clinicians to rapidly diagnose identify and utilize monovalent antivenom to undo the serious consequences of snake bite. At the same time, we are aware that using metabolomics alone to find key metabolites in snakebite is still far from adequate, and that differences in identification of some metabolites may cause us to miss some metabolites that may be important in snakebite due to limitations in instrumentation and technology, and that we may need more clinical samples to verify whether the differential metabolites we obtained are widespread.

## Conclusions

In this study, a *Bama miniature pig* model of *Naja atra* bite was successfully established. Through untargeted metabolomics and targeted metabolomics, we found that glutamine, arginine, proline, leucine, phenylalanine, Inosine, thymidine and hippuric acid may be potential biomarkers of *Naja atra* poisoning. Validation of serum samples from clinical *Naja atra* bite patients indicated that inosine, proline and phenylalanine are expected to be key metabolites for the identification of *Naja atra* bite. At the same time, through the functional analysis of the core metabolites, we expounded the pathophysiological mechanism of *Naja atra* bite and the complex interaction between the changes of *Naja atra* bite metabolites and the body’s response from the perspective of metabolism, in order to provide a basis for the clinical treatment of *Naja atra* bite.

## Supporting information

S1 Fig TIC plot of serum samples. (A)Venom group in ESI+ (B) Control group in ESI+ (C) Venom group in ESI- (D) Control group in ESI-.
(DOCX)

S2 Fig Standard curve of different metabolite standards with different concentration gradients. (A)Inosine, (B)Hippuric acid, (C)Glycochenodeoxycholate, (D)Thymidine, (E)Glutamine, (F)Leucine, (G)Phenylalanine, (H)Proline, (I)Arginine.
(DOCX)

S3 Fig Correlation spectrum of QC samples in ESI+ and ESI- mode. A, ESI+ B, ESI-.
(DOCX)

S1 Table List of differential metabolites in ESI+. (XLSX)

S2 Table List of differential metabolites in ESI-. (XLSX)
