# Supplementary material for: Metabolomics analyses of serum metabolites perturbations associated with Naja atra bite
Source: PLoS Negl Trop Dis. 2023 Aug 28;17(8):e0011507. doi: 10.1371/journal.pntd.0011507 (PMC10461852; doi:10.1371/journal.pntd.0011507)
Supplement: S1 Fig — (A)Venom group in ESI+ (B) Control group in ESI+ (C) Venom group in ESI- (D) Control group in ESI-. (DOCX) [file pntd.0011507.s001.docx]

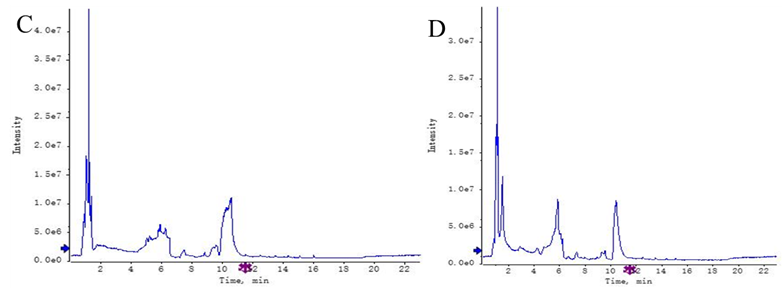

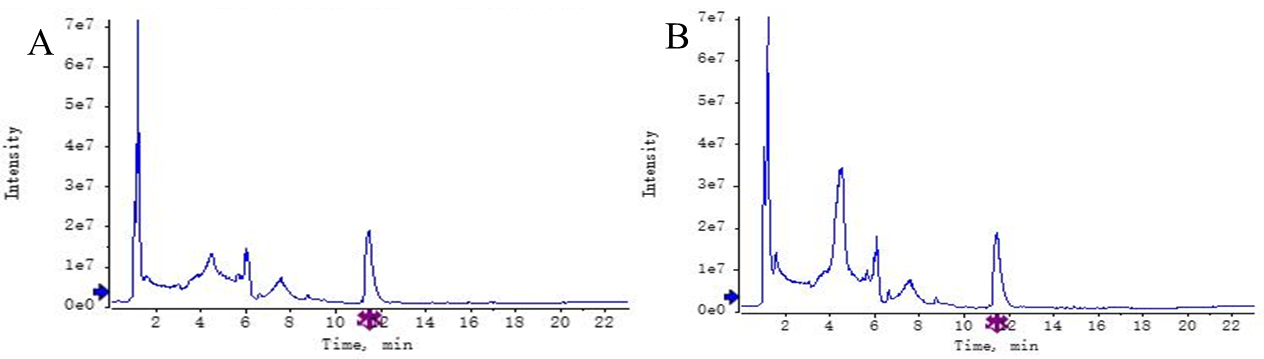
S1 TIC plot of serum samples. (A)Venom group in ESI+ (B) Control group in ESI+ (C) Venom group in ESI- (D) Control group in ESI-.
