# Supplementary material for: Metabolomics analyses of serum metabolites perturbations associated with Naja atra bite
Source: PLoS Negl Trop Dis. 2023 Aug 28;17(8):e0011507. doi: 10.1371/journal.pntd.0011507 (PMC10461852; doi:10.1371/journal.pntd.0011507)
Supplement: S2 Fig — (A)Inosine, (B)Hippuric acid, (C)Glycochenodeoxycholate, (D)Thymidine, (E)Glutamine, (F)Leucine, (G)Phenylalanine, (H)Proline, (I)Arginine. (DOCX) [file pntd.0011507.s002.docx]

**
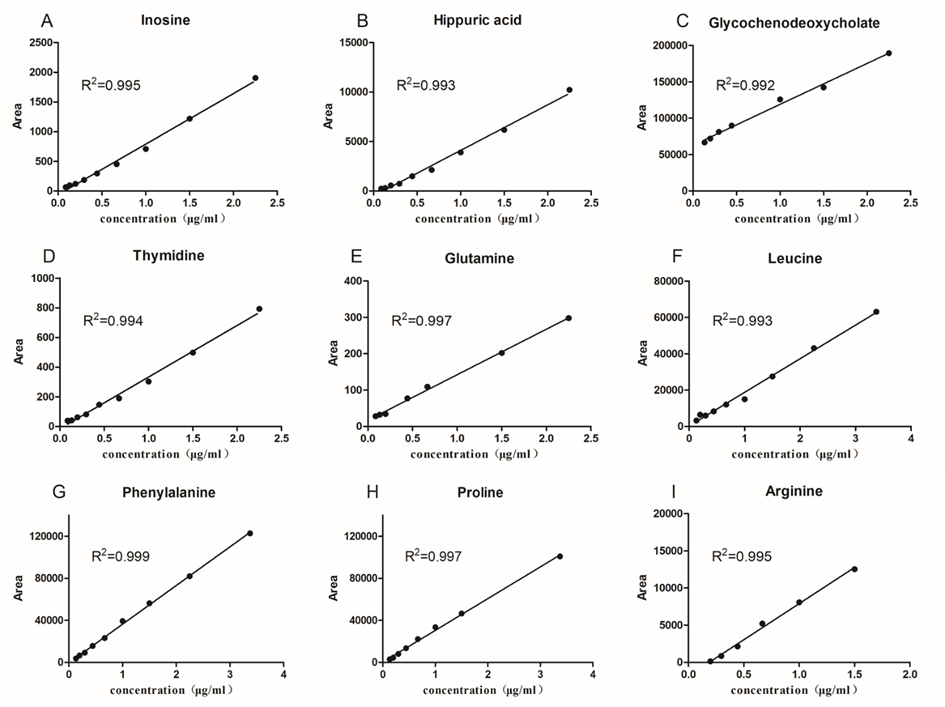
**

**S2** Standard curve of different metabolite standards with different concentration gradients.(A)Inosine, (B)Hippuric acid, (C)Glycochenodeoxycholate, (D)Thymidine, (E)Glutamine, (F)Leucine, (G)Phenylalanine, (H)Proline, (I)Arginine.
