# Supplementary figures and images for: Metabolomics analyses of serum metabolites perturbations associated with Naja atra bite
Source: PLoS Negl Trop Dis. 2023 Aug 28;17(8):e0011507. doi: 10.1371/journal.pntd.0011507 (PMC10461852; doi:10.1371/journal.pntd.0011507)

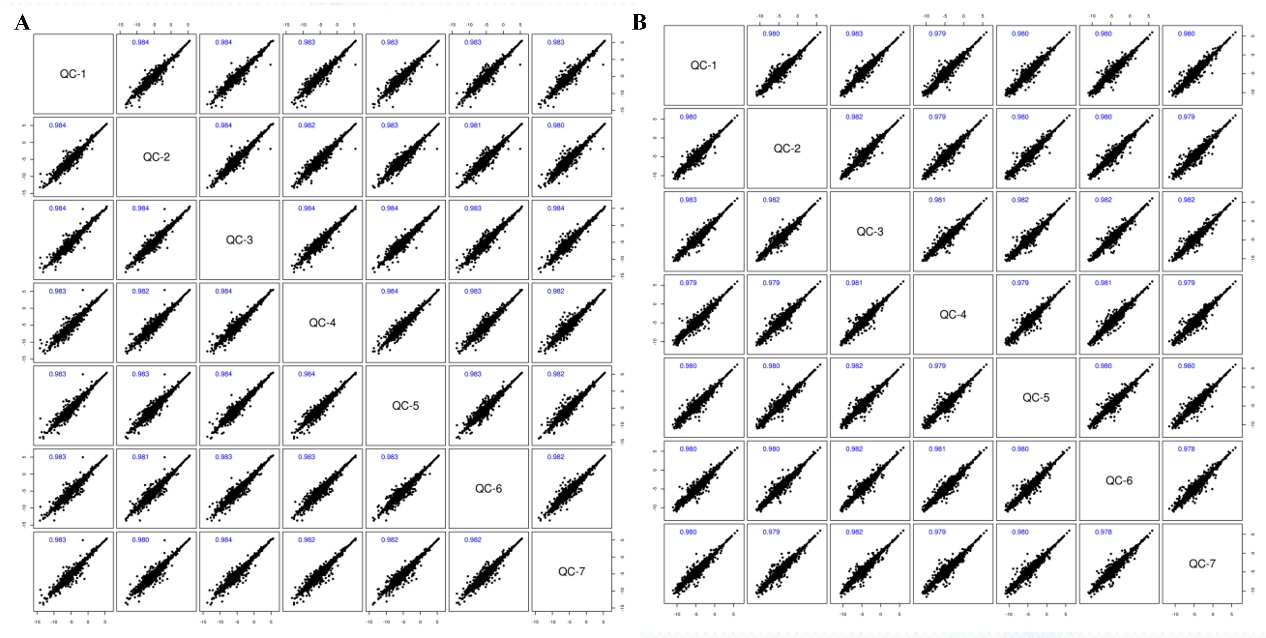
S3：Correlation spectrum of QC samples in ESI+ and ESI- mode. A,ESI+ B,ESI-

Supplement: S3 Fig — A, ESI+ B, ESI-. (DOCX) [file pntd.0011507.s003.docx]
